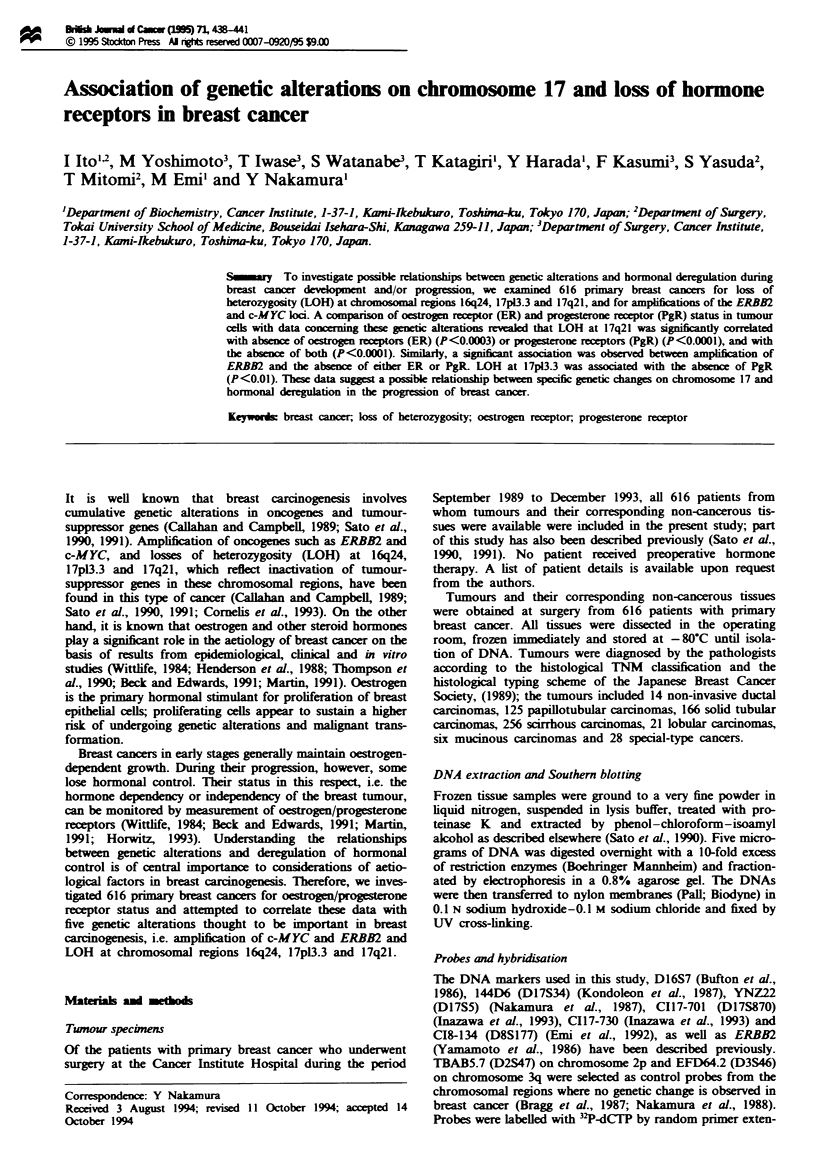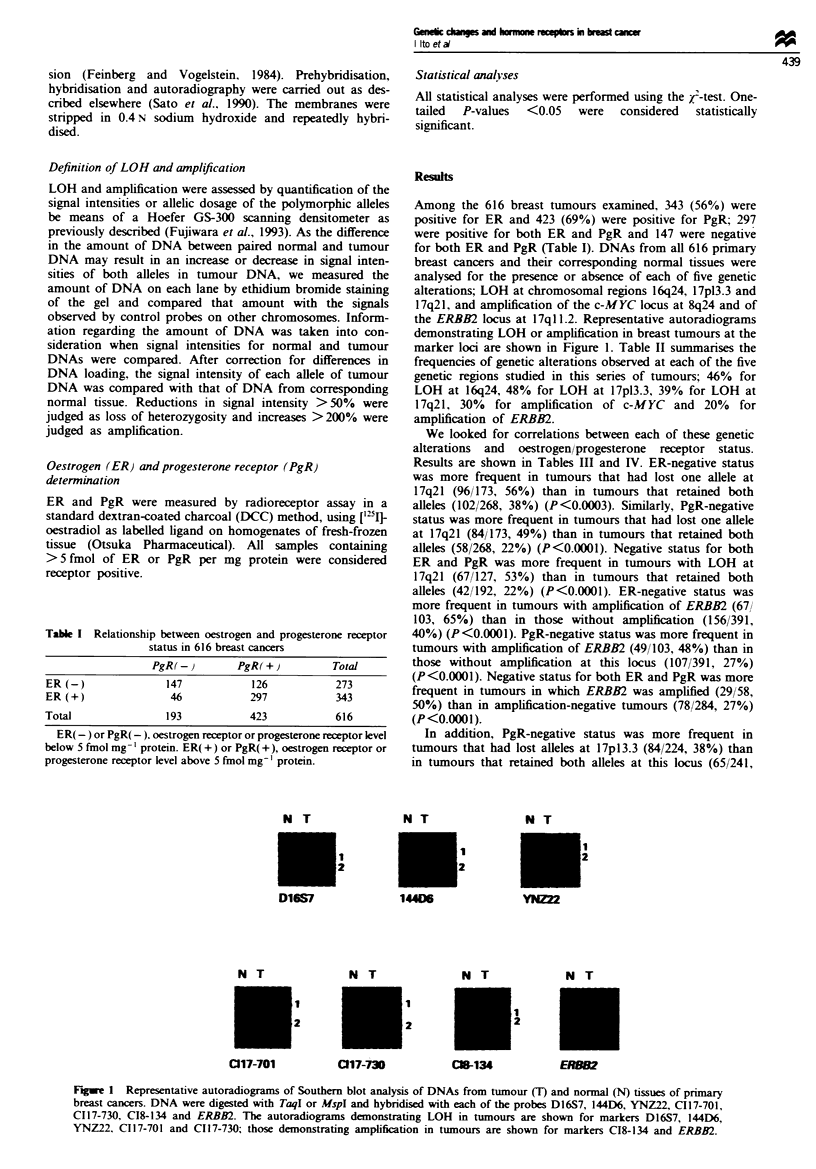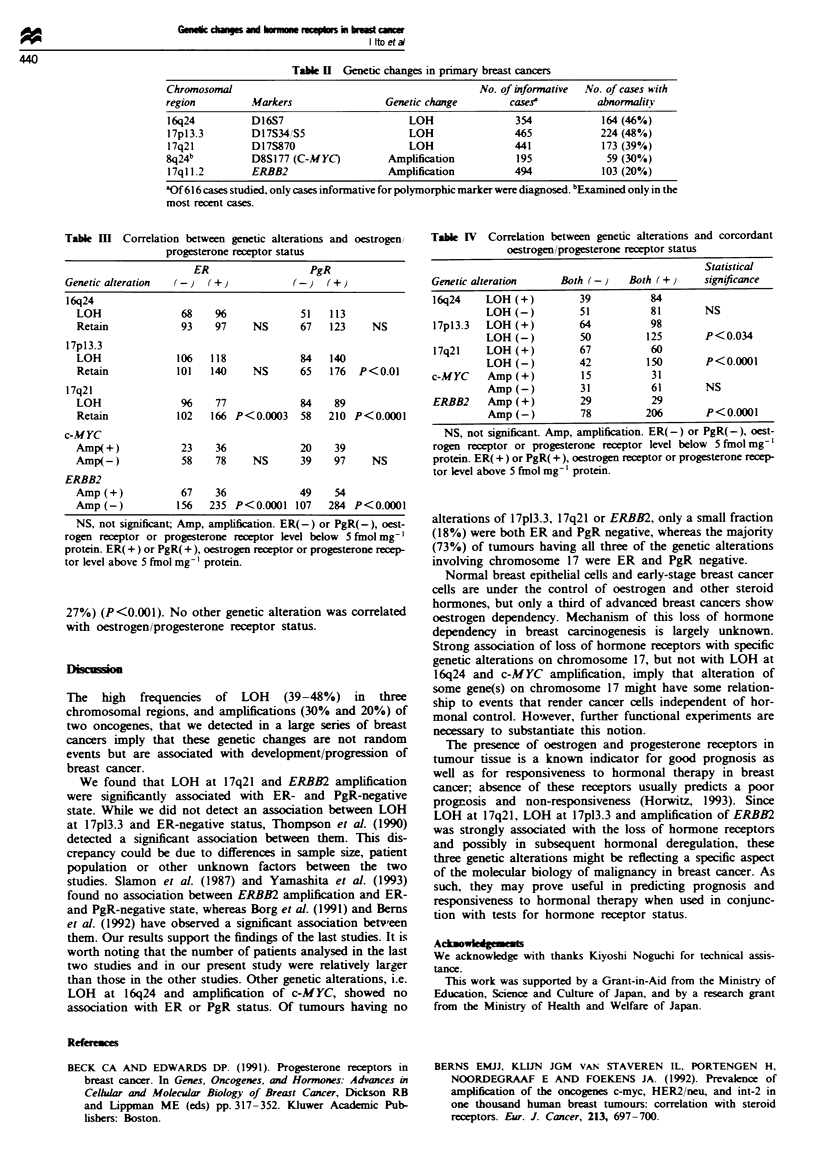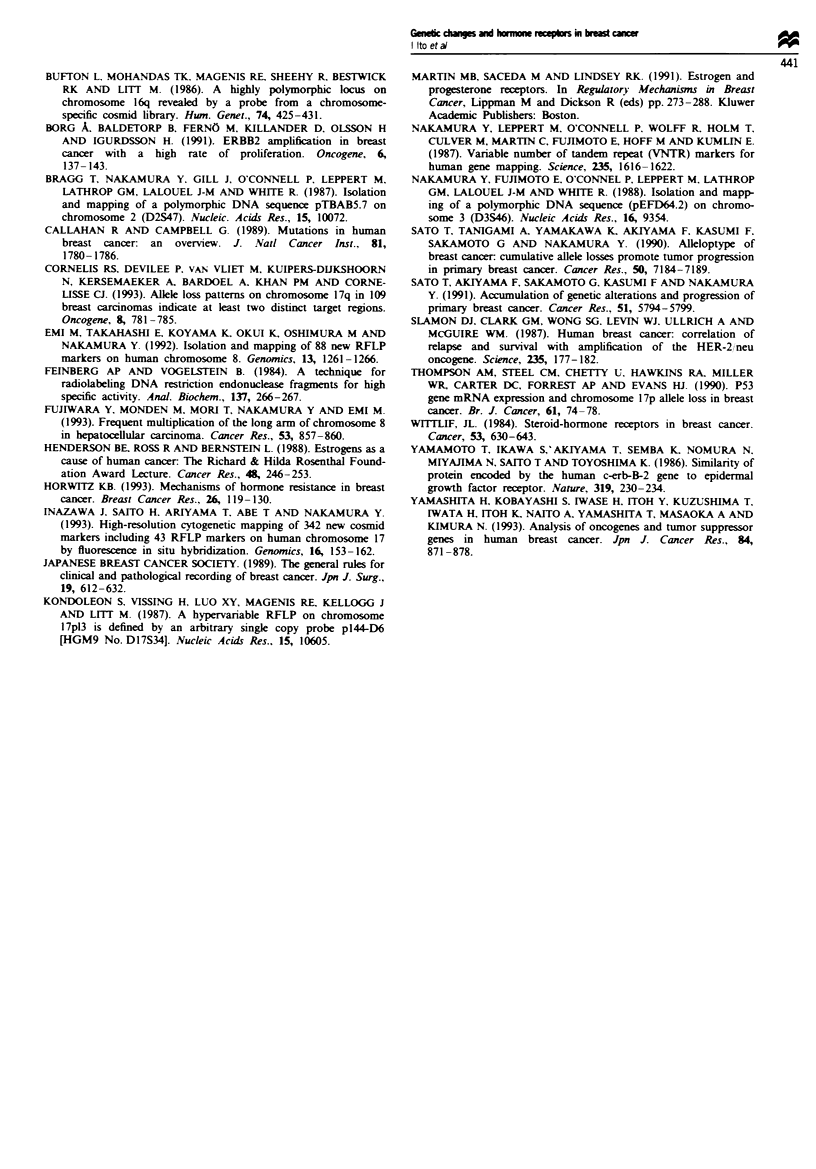# Association of genetic alterations on chromosome 17 and loss of hormone receptors in breast cancer.

**DOI:** 10.1038/bjc.1995.89

**Published:** 1995-03

**Authors:** I. Ito, M. Yoshimoto, T. Iwase, S. Watanabe, T. Katagiri, Y. Harada, F. Kasumi, S. Yasuda, T. Mitomi, M. Emi

**Affiliations:** Department of Biochemistry, Cancer Institute, Tokyo, Japan.

## Abstract

**Images:**


© 1995 Stockton Press All rights reserved 0007–0920/95 $9.00

### Association of genetic alterations on chromosome 17 and loss of hormone receptors in breast cancer

I Ito[1,2], M Yoshimoto[3], T Iwase[3], S Watanabe[3], T Katagiri[1], Y Harada[1], F Kasumi[3], S Yasuda[2], T Mitomi[2], M Emi[1] and Y Nakamura[1]

[1]*Department of Biochemistry, Cancer Institute, 1-37-1, Kami-Ikebukuro, Toshima-ku, Tokyo 170, Japan;* [2]*Department of Surgery, Tokai University School of Medicine, Bouseidai Isehara-Shi, Kanagawa 259-11, Japan;* [3]*Department of Surgery, Cancer Institute, 1-37-1, Kami-Ikebukuro, Toshima-ku, Tokyo 170, Japan.*

**Summary** To investigate possible relationships between genetic alterations and hormonal deregulation during breast cancer development and/or progression, we examined 616 primary breast cancers for loss of heterozygosity (LOH) at chromosomal regions 16q24, 17p13.3 and 17q21, and for amplifications of the *ERBB2* and c-*MYC* loci. A comparison of oestrogen receptor (ER) and progesterone receptor (PgR) status in tumour cells with data concerning these genetic alterations revealed that LOH at 17q21 was significantly correlated with absence of oestrogen receptors (ER) ($P<0.0003$) or progesterone receptors (PgR) ($P<0.0001$), and with the absence of both ($P<0.0001$). Similarly, a significant association was observed between amplification of *ERBB2* and the absence of either ER or PgR. LOH at 17p13.3 was associated with the absence of PgR ($P<0.01$). These data suggest a possible relationship between specific genetic changes on chromosome 17 and hormonal deregulation in the progression of breast cancer.

**Keywords:** breast cancer; loss of heterozygosity; oestrogen receptor; progesterone receptor

It is well known that breast carcinogenesis involves cumulative genetic alterations in oncogenes and tumour-suppressor genes (Callahan and Campbell, 1989; Sato *et al.*, 1990, 1991). Amplification of oncogenes such as *ERBB2* and c-*MYC*, and losses of heterozygosity (LOH) at 16q24, 17p13.3 and 17q21, which reflect inactivation of tumour-suppressor genes in these chromosomal regions, have been found in this type of cancer (Callahan and Campbell, 1989; Sato *et al.*, 1990, 1991; Cornelis *et al.*, 1993). On the other hand, it is known that oestrogen and other steroid hormones play a significant role in the aetiology of breast cancer on the basis of results from epidemiological, clinical and *in vitro* studies (Wittlife, 1984; Henderson *et al.*, 1988; Thompson *et al.*, 1990; Beck and Edwards, 1991; Martin, 1991). Oestrogen is the primary hormonal stimulant for proliferation of breast epithelial cells; proliferating cells appear to sustain a higher risk of undergoing genetic alterations and malignant transformation.

Breast cancers in early stages generally maintain oestrogen-dependent growth. During their progression, however, some lose hormonal control. Their status in this respect, i.e. the hormone dependency or independency of the breast tumour, can be monitored by measurement of oestrogen/progesterone receptors (Wittlife, 1984; Beck and Edwards, 1991; Martin, 1991; Horwitz, 1993). Understanding the relationships between genetic alterations and deregulation of hormonal control is of central importance to considerations of aetiological factors in breast carcinogenesis. Therefore, we investigated 616 primary breast cancers for oestrogen/progesterone receptor status and attempted to correlate these data with five genetic alterations thought to be important in breast carcinogenesis, i.e. amplification of c-*MYC* and *ERBB2* and LOH at chromosomal regions 16q24, 17p13.3 and 17q21.

### Materials and methods

### *Tumour specimens*

Of the patients with primary breast cancer who underwent surgery at the Cancer Institute Hospital during the period September 1989 to December 1993, all 616 patients from whom tumours and their corresponding non-cancerous tissues were available were included in the present study; part of this study has also been described previously (Sato *et al.*, 1990, 1991). No patient received preoperative hormone therapy. A list of patient details is available upon request from the authors.

Tumours and their corresponding non-cancerous tissues were obtained at surgery from 616 patients with primary breast cancer. All tissues were dissected in the operating room, frozen immediately and stored at −80°C until isolation of DNA. Tumours were diagnosed by the pathologists according to the histological TNM classification and the histological typing scheme of the Japanese Breast Cancer Society, (1989); the tumours included 14 non-invasive ductal carcinomas, 125 papillotubular carcinomas, 166 solid tubular carcinomas, 256 scirrhous carcinomas, 21 lobular carcinomas, six mucinous carcinomas and 28 special-type cancers.

### *DNA extraction and Southern blotting*

Frozen tissue samples were ground to a very fine powder in liquid nitrogen, suspended in lysis buffer, treated with proteinase K and extracted by phenol–chloroform–isoamyl alcohol as described elsewhere (Sato *et al.*, 1990). Five micrograms of DNA was digested overnight with a 10-fold excess of restriction enzymes (Boehringer Mannheim) and fractionated by electrophoresis in a 0.8% agarose gel. The DNAs were then transferred to nylon membranes (Pall; Biodyne) in 0.1 N sodium hydroxide–0.1 M sodium chloride and fixed by UV cross-linking.

### *Probes and hybridisation*

The DNA markers used in this study, D16S7 (Bufton *et al.*, 1986), 144D6 (D17S34) (Kondoleon *et al.*, 1987), YNZ22 (D17S5) (Nakamura *et al.*, 1987), CI17-701 (D17S870) (Inazawa *et al.*, 1993), CI17-730 (Inazawa *et al.*, 1993) and CI8-134 (D8S177) (Emi *et al.*, 1992), as well as *ERBB2* (Yamamoto *et al.*, 1986) have been described previously. TBAB5.7 (D2S47) on chromosome 2p and EFD64.2 (D3S46) on chromosome 3q were selected as control probes from the chromosomal regions where no genetic change is observed in breast cancer (Bragg *et al.*, 1987; Nakamura *et al.*, 1988). Probes were labelled with $^{32}$P-dCTP by random primer exten-

Correspondence: Y Nakamura
Received 3 August 1994; revised 11 October 1994; accepted 14 October 1994

sion (Feinberg and Vogelstein, 1984). Prehybridisation, hybridisation and autoradiography were carried out as described elsewhere (Sato *et al.*, 1990). The membranes were stripped in 0.4 N sodium hydroxide and repeatedly hybridised.

*Definition of LOH and amplification*

LOH and amplification were assessed by quantification of the signal intensities or allelic dosage of the polymorphic alleles be means of a Hoefer GS-300 scanning densitometer as previously described (Fujiwara *et al.*, 1993). As the difference in the amount of DNA between paired normal and tumour DNA may result in an increase or decrease in signal intensities of both alleles in tumour DNA, we measured the amount of DNA on each lane by ethidium bromide staining of the gel and compared that amount with the signals observed by control probes on other chromosomes. Information regarding the amount of DNA was taken into consideration when signal intensities for normal and tumour DNAs were compared. After correction for differences in DNA loading, the signal intensity of each allele of tumour DNA was compared with that of DNA from corresponding normal tissue. Reductions in signal intensity >50% were judged as loss of heterozygosity and increases >200% were judged as amplification.

*Oestrogen (ER) and progesterone receptor (PgR) determination*

ER and PgR were measured by radioreceptor assay in a standard dextran-coated charcoal (DCC) method, using [$^{125}$I]-oestradiol as labelled ligand on homogenates of fresh-frozen tissue (Otsuka Pharmaceutical). All samples containing >5 fmol of ER or PgR per mg protein were considered receptor positive.

**Table I** Relationship between oestrogen and progesterone receptor status in 616 breast cancers

| | *PgR(−)* | *PgR(+)* | *Total* |
|---|---|---|---|
| ER (−) | 147 | 126 | 273 |
| ER (+) | 46 | 297 | 343 |
| Total | 193 | 423 | 616 |

ER(−) or PgR(−), oestrogen receptor or progesterone receptor level below 5 fmol mg$^{-1}$ protein. ER(+) or PgR(+), oestrogen receptor or progesterone receptor level above 5 fmol mg$^{-1}$ protein.

*Statistical analyses*

All statistical analyses were performed using the $\chi^2$-test. One-tailed *P*-values <0.05 were considered statistically significant.

### Results

Among the 616 breast tumours examined, 343 (56%) were positive for ER and 423 (69%) were positive for PgR; 297 were positive for both ER and PgR and 147 were negative for both ER and PgR (Table I). DNAs from all 616 primary breast cancers and their corresponding normal tissues were analysed for the presence or absence of each of five genetic alterations; LOH at chromosomal regions 16q24, 17p13.3 and 17q21, and amplification of the c-*MYC* locus at 8q24 and of the *ERBB2* locus at 17q11.2. Representative autoradiograms demonstrating LOH or amplification in breast tumours at the marker loci are shown in Figure 1. Table II summarises the frequencies of genetic alterations observed at each of the five genetic regions studied in this series of tumours; 46% for LOH at 16q24, 48% for LOH at 17p13.3, 39% for LOH at 17q21, 30% for amplification of c-*MYC* and 20% for amplification of *ERBB2*.

We looked for correlations between each of these genetic alterations and oestrogen/progesterone receptor status. Results are shown in Tables III and IV. ER-negative status was more frequent in tumours that had lost one allele at 17q21 (96/173, 56%) than in tumours that retained both alleles (102/268, 38%) ($P<0.0003$). Similarly, PgR-negative status was more frequent in tumours that had lost one allele at 17q21 (84/173, 49%) than in tumours that retained both alleles (58/268, 22%) ($P<0.0001$). Negative status for both ER and PgR was more frequent in tumours with LOH at 17q21 (67/127, 53%) than in tumours that retained both alleles (42/192, 22%) ($P<0.0001$). ER-negative status was more frequent in tumours with amplification of *ERBB2* (67/103, 65%) than in those without amplification (156/391, 40%) ($P<0.0001$). PgR-negative status was more frequent in tumours with amplification of *ERBB2* (49/103, 48%) than in those without amplification at this locus (107/391, 27%) ($P<0.0001$). Negative status for both ER and PgR was more frequent in tumours in which *ERBB2* was amplified (29/58, 50%) than in amplification-negative tumours (78/284, 27%) ($P<0.0001$).

In addition, PgR-negative status was more frequent in tumours that had lost alleles at 17p13.3 (84/224, 38%) than in tumours that retained both alleles at this locus (65/241,

**Figure 1** Representative autoradiograms of Southern blot analysis of DNAs from tumour (T) and normal (N) tissues of primary breast cancers. DNA were digested with *Taq*I or *Msp*I and hybridised with each of the probes D16S7, 144D6, YNZ22, CI17-701, CI17-730, CI8-134 and *ERBB2*. The autoradiograms demonstrating LOH in tumours are shown for markers D16S7, 144D6, YNZ22, CI17-701 and CI17-730; those demonstrating amplification in tumours are shown for markers CI8-134 and *ERBB2*.

**Table II** Genetic changes in primary breast cancers

| *Chromosomal region* | *Markers* | *Genetic change* | *No. of informative cases*[a] | *No. of cases with abnormality* |
|---|---|---|---|---|
| 16q24 | D16S7 | LOH | 354 | 164 (46%) |
| 17p13.3 | D17S34/S5 | LOH | 465 | 224 (48%) |
| 17q21 | D17S870 | LOH | 441 | 173 (39%) |
| 8q24[b] | D8S177 (C-*MYC*) | Amplification | 195 | 59 (30%) |
| 17q11.2 | *ERBB2* | Amplification | 494 | 103 (20%) |

[a]Of 616 cases studied, only cases informative for polymorphic marker were diagnosed. [b]Examined only in the most recent cases.

**Table III** Correlation between genetic alterations and oestrogen/progesterone receptor status

| *Genetic alteration* | *ER* (−) | *ER* (+) | | *PgR* (−) | *PgR* (+) | |
|---|---|---|---|---|---|---|
| 16q24 | | | | | | |
| LOH | 68 | 96 | | 51 | 113 | |
| Retain | 93 | 97 | NS | 67 | 123 | NS |
| 17p13.3 | | | | | | |
| LOH | 106 | 118 | | 84 | 140 | |
| Retain | 101 | 140 | NS | 65 | 176 | $P<0.01$ |
| 17q21 | | | | | | |
| LOH | 96 | 77 | | 84 | 89 | |
| Retain | 102 | 166 | $P<0.0003$ | 58 | 210 | $P<0.0001$ |
| c-*MYC* | | | | | | |
| Amp(+) | 23 | 36 | | 20 | 39 | |
| Amp(−) | 58 | 78 | NS | 39 | 97 | NS |
| *ERBB2* | | | | | | |
| Amp (+) | 67 | 36 | | 49 | 54 | |
| Amp (−) | 156 | 235 | $P<0.0001$ | 107 | 284 | $P<0.0001$ |

NS, not significant; Amp, amplification. ER(−) or PgR(−), oestrogen receptor or progesterone receptor level below 5 fmol $mg^{-1}$ protein. ER(+) or PgR(+), oestrogen receptor or progesterone receptor level above 5 fmol $mg^{-1}$ protein.

**Table IV** Correlation between genetic alterations and corcordant oestrogen/progesterone receptor status

| *Genetic alteration* | | *Both (−)* | *Both (+)* | *Statistical significance* |
|---|---|---|---|---|
| 16q24 | LOH (+) | 39 | 84 | |
| | LOH (−) | 51 | 81 | NS |
| 17p13.3 | LOH (+) | 64 | 98 | |
| | LOH (−) | 50 | 125 | $P<0.034$ |
| 17q21 | LOH (+) | 67 | 60 | |
| | LOH (−) | 42 | 150 | $P<0.0001$ |
| c-*MYC* | Amp (+) | 15 | 31 | |
| | Amp (−) | 31 | 61 | NS |
| *ERBB2* | Amp (+) | 29 | 29 | |
| | Amp (−) | 78 | 206 | $P<0.0001$ |

NS, not significant. Amp, amplification. ER(−) or PgR(−), oestrogen receptor or progesterone receptor level below 5 fmol $mg^{-1}$ protein. ER(+) or PgR(+), oestrogen receptor or progesterone receptor level above 5 fmol $mg^{-1}$ protein.

27%) ($P<0.001$). No other genetic alteration was correlated with oestrogen/progesterone receptor status.

### Discussion

The high frequencies of LOH (39–48%) in three chromosomal regions, and amplifications (30% and 20%) of two oncogenes, that we detected in a large series of breast cancers imply that these genetic changes are not random events but are associated with development/progression of breast cancer.

We found that LOH at 17q21 and *ERBB2* amplification were significantly associated with ER- and PgR-negative state. While we did not detect an association between LOH at 17pl3.3 and ER-negative status, Thompson *et al.* (1990) detected a significant association between them. This discrepancy could be due to differences in sample size, patient population or other unknown factors between the two studies. Slamon *et al.* (1987) and Yamashita *et al.* (1993) found no association between *ERBB2* amplification and ER- and PgR-negative state, whereas Borg *et al.* (1991) and Berns *et al.* (1992) have observed a significant association between them. Our results support the findings of the last studies. It is worth noting that the number of patients analysed in the last two studies and in our present study were relatively larger than those in the other studies. Other genetic alterations, i.e. LOH at 16q24 and amplification of c-*MYC*, showed no association with ER or PgR status. Of tumours having no alterations of 17pl3.3, 17q21 or *ERBB2*, only a small fraction (18%) were both ER and PgR negative, whereas the majority (73%) of tumours having all three of the genetic alterations involving chromosome 17 were ER and PgR negative.

Normal breast epithelial cells and early-stage breast cancer cells are under the control of oestrogen and other steroid hormones, but only a third of advanced breast cancers show oestrogen dependency. Mechanism of this loss of hormone dependency in breast carcinogenesis is largely unknown. Strong association of loss of hormone receptors with specific genetic alterations on chromosome 17, but not with LOH at 16q24 and c-*MYC* amplification, imply that alteration of some gene(s) on chromosome 17 might have some relationship to events that render cancer cells independent of hormonal control. However, further functional experiments are necessary to substantiate this notion.

The presence of oestrogen and progesterone receptors in tumour tissue is a known indicator for good prognosis as well as for responsiveness to hormonal therapy in breast cancer; absence of these receptors usually predicts a poor prognosis and non-responsiveness (Horwitz, 1993). Since LOH at 17q21, LOH at 17pl3.3 and amplification of *ERBB2* was strongly associated with the loss of hormone receptors and possibly in subsequent hormonal deregulation, these three genetic alterations might be reflecting a specific aspect of the molecular biology of malignancy in breast cancer. As such, they may prove useful in predicting prognosis and responsiveness to hormonal therapy when used in conjunction with tests for hormone receptor status.

**Acknowledgements**

We acknowledge with thanks Kiyoshi Noguchi for technical assistance.

This work was supported by a Grant-in-Aid from the Ministry of Education, Science and Culture of Japan, and by a research grant from the Ministry of Health and Welfare of Japan.

### References

BECK CA AND EDWARDS DP. (1991). Progesterone receptors in breast cancer. In *Genes, Oncogenes, and Hormones: Advances in Cellular and Molecular Biology of Breast Cancer*, Dickson RB and Lippman ME (eds) pp. 317–352. Kluwer Academic Publishers: Boston.

BERNS EMJJ, KLIJN JGM VAN STAVEREN IL, PORTENGEN H, NOORDEGRAAF E AND FOEKENS JA. (1992). Prevalence of amplification of the oncogenes c-myc, HER2/neu, and int-2 in one thousand human breast tumours: correlation with steroid receptors. *Eur. J. Cancer*, **213**, 697–700.

BUFTON L, MOHANDAS TK, MAGENIS RE, SHEEHY R, BESTWICK RK AND LITT M. (1986). A highly polymorphic locus on chromosome 16q revealed by a probe from a chromosome-specific cosmid library. *Hum. Genet.*, **74,** 425–431.

BORG Å, BALDETORP B, FERNÖ M, KILLANDER D, OLSSON H AND IGURDSSON H. (1991). ERBB2 amplification in breast cancer with a high rate of proliferation. *Oncogene*, **6,** 137–143.

BRAGG T, NAKAMURA Y, GILL J, O'CONNELL P, LEPPERT M, LATHROP GM, LALOUEL J-M AND WHITE R. (1987). Isolation and mapping of a polymorphic DNA sequence pTBAB5.7 on chromosome 2 (D2S47). *Nucleic. Acids Res.*, **15,** 10072.

CALLAHAN R AND CAMPBELL G. (1989). Mutations in human breast cancer: an overview. *J. Natl Cancer Inst.*, **81,** 1780–1786.

CORNELIS RS, DEVILEE P, VAN VLIET M, KUIPERS-DIJKSHOORN N, KERSEMAEKER A, BARDOEL A, KHAN PM AND CORNELISSE CJ. (1993). Allele loss patterns on chromosome 17q in 109 breast carcinomas indicate at least two distinct target regions. *Oncogene*, **8,** 781–785.

EMI M, TAKAHASHI E, KOYAMA K, OKUI K, OSHIMURA M AND NAKAMURA Y. (1992). Isolation and mapping of 88 new RFLP markers on human chromosome 8. *Genomics*, **13,** 1261–1266.

FEINBERG AP AND VOGELSTEIN B. (1984). A technique for radiolabeling DNA restriction endonuclease fragments for high specific activity. *Anal. Biochem.*, **137,** 266–267.

FUJIWARA Y, MONDEN M, MORI T, NAKAMURA Y AND EMI M. (1993). Frequent multiplication of the long arm of chromosome 8 in hepatocellular carcinoma. *Cancer Res.*, **53,** 857–860.

HENDERSON BE, ROSS R AND BERNSTEIN L. (1988). Estrogens as a cause of human cancer: The Richard & Hilda Rosenthal Foundation Award Lecture. *Cancer Res.*, **48,** 246–253.

HORWITZ KB. (1993). Mechanisms of hormone resistance in breast cancer. *Breast Cancer Res.*, **26,** 119–130.

INAZAWA J, SAITO H, ARIYAMA T, ABE T AND NAKAMURA Y. (1993). High-resolution cytogenetic mapping of 342 new cosmid markers including 43 RFLP markers on human chromosome 17 by fluorescence in situ hybridization. *Genomics*, **16,** 153–162.

JAPANESE BREAST CANCER SOCIETY. (1989). The general rules for clinical and pathological recording of breast cancer. *Jpn J. Surg.*, **19,** 612–632.

KONDOLEON S, VISSING H, LUO XY, MAGENIS RE, KELLOGG J AND LITT M. (1987). A hypervariable RFLP on chromosome 17pl3 is defined by an arbitrary single copy probe pl44-D6 [HGM9 No. D17S34]. *Nucleic Acids Res.*, **15,** 10605.

MARTIN MB, SACEDA M AND LINDSEY RK. (1991). Estrogen and progesterone receptors. In *Regulatory Mechanisms in Breast Cancer*, Lippman M and Dickson R (eds) pp. 273–288. Kluwer Academic Publishers: Boston.

NAKAMURA Y, LEPPERT M, O'CONNELL P, WOLFF R, HOLM T, CULVER M, MARTIN C, FUJIMOTO E, HOFF M AND KUMLIN E. (1987). Variable number of tandem repeat (VNTR) markers for human gene mapping. *Science*, **235,** 1616–1622.

NAKAMURA Y, FUJIMOTO E, O'CONNEL P, LEPPERT M, LATHROP GM, LALOUEL J-M AND WHITE R. (1988). Isolation and mapping of a polymorphic DNA sequence (pEFD64.2) on chromosome 3 (D3S46). *Nucleic Acids Res.*, **16,** 9354.

SATO T, TANIGAMI A, YAMAKAWA K, AKIYAMA F, KASUMI F, SAKAMOTO G AND NAKAMURA Y. (1990). Alleloptype of breast cancer: cumulative allele losses promote tumor progression in primary breast cancer. *Cancer Res.*, **50,** 7184–7189.

SATO T, AKIYAMA F, SAKAMOTO G, KASUMI F AND NAKAMURA Y. (1991). Accumulation of genetic alterations and progression of primary breast cancer. *Cancer Res.*, **51,** 5794–5799.

SLAMON DJ, CLARK GM, WONG SG, LEVIN WJ, ULLRICH A AND McGUIRE WM. (1987). Human breast cancer: correlation of relapse and survival with amplification of the HER-2/neu oncogene. *Science*, **235,** 177–182.

THOMPSON AM, STEEL CM, CHETTY U, HAWKINS RA, MILLER WR, CARTER DC, FORREST AP AND EVANS HJ. (1990). P53 gene mRNA expression and chromosome 17p allele loss in breast cancer. *Br. J. Cancer*, **61,** 74–78.

WITTLIF, JL. (1984). Steroid-hormone receptors in breast cancer. *Cancer*, **53,** 630–643.

YAMAMOTO T, IKAWA S,`AKIYAMA T, SEMBA K, NOMURA N, MIYAJIMA N, SAITO T AND TOYOSHIMA K. (1986). Similarity of protein encoded by the human c-erb-B-2 gene to epidermal growth factor receptor. *Nature*, **319,** 230–234.

YAMASHITA H, KOBAYASHI S, IWASE H, ITOH Y, KUZUSHIMA T, IWATA H, ITOH K, NAITO A, YAMASHITA T, MASAOKA A AND KIMURA N. (1993). Analysis of oncogenes and tumor suppressor genes in human breast cancer. *Jpn J. Cancer Res.*, **84,** 871–878.